# Regenerative Polarity of the Fin Ray in Zebrafish Caudal Fin and Related Tissue Formation on the Cut Surface

**DOI:** 10.3390/jdb9040050

**Published:** 2021-11-19

**Authors:** Wataru Nakajima, Soya Nakanishi, Ryosuke Hosoya, Toshiaki Uemoto, Shiro Ohgo, Naoyuki Wada

**Affiliations:** Department of Applied Biological Science, Tokyo University of Science, 2641 Yamazaki, Noda 278-8510, Japan; sobakarute_sub@yahoo.co.jp (W.N.); nakanishisoya@gmail.com (S.N.); masha-guiter.pc@outlook.com (R.H.); shimashima0401fish@gmail.com (T.U.); ohgo@kitasato-u.ac.jp (S.O.)

**Keywords:** zebrafish, fin regeneration, tissue polarity, wound epithelium

## Abstract

Zebrafish caudal fin rays are used as a model system for regeneration because of their high regenerative ability, but studies on the regeneration polarity of the fin ray are limited. To investigate this regeneration polarity, we made a hole to excise part of the fin ray and analyzed the regeneration process. We confirmed that the fin rays always regenerated from the proximal margin toward the distal margin, as previously reported; however, regeneration-related genes were expressed at both the proximal and distal edges of the hole in the early stage of regeneration, suggesting that the regenerative response also occurs at the distal edge. One difference between the proximal and distal margins is a sheet-like tissue that is formed on the apical side of the regenerated tissue at the proximal margin. This sheet-like tissue was not observed at the distal edge. To investigate whether the distal margin was also capable of forming this sheet-like tissue and subsequent regeneration, we kept the distal margin separated from the proximal margin by manipulation. Consequently, the sheet-like tissue was formed at the distal margin and regeneration of the fin ray was also induced. The regenerated fin rays from the distal margin protruded laterally from the caudal fin and then bent distally, and their ends showed the same characteristics as those of the normal fin rays. These results suggest that fin rays have an ability to regenerate in both directions; however, under normal conditions, regeneration is restricted to the proximal margin because the sheet-like tissue is preferentially formed on the apical side of the regenerating tissue from the proximal margin.

## 1. Introduction

Zebrafish have a high regenerative capacity in many organs, including the retina, spinal cord, part of the heart, and fins [1,2,3,4]. In particular, the caudal fin is often used as a model system for regeneration studies (for review see [3,4,5,6,7,8,9]). The caudal fin skeleton of zebrafish is composed of bony tissues called fin rays, and each fin ray consists of two semicircular structures called hemi-rays. Fin rays are connected by an epithelial sheet with connective tissue, which is called the inter-ray [7,8,10].

Regeneration proceeds in the following three stages: wound healing, blastema formation, and tissue reorganization [8,11]. In the wound healing phase, the cut surface is closed and covered by an epithelial tissue called the wound epithelium [12,13]. Then, the wound epithelium secretes diffusible signaling molecules, and these molecules, in turn, induce the cells beneath the epithelium to dedifferentiate and form the blastema. The blastema is composed of undifferentiated mesenchymal cells beneath the wound epithelium and elongates distally by active proliferation of the mesenchymal cells. In parallel, cells in the proximal region of the blastema begin to re-differentiate into bone and surrounding connective tissue [5,6,9]. Analysis of the gene expression and molecular interactions during the fin regeneration has revealed genes that are selectively expressed in the wound epithelium or regeneration blastema [6,7,9,14,15,16,17]. For example, *fgf24*, its downstream factor *pea3*, and the transcription factor *msxd* are expressed in the wound epithelium from an early stage of regeneration [18,19,20,21,22]. Moreover, expression of genes such as *msxb* and *fgf20a* has been reported in mesenchymal cells, which constitute the blastema [21,22,23,24]. The expression of these genes can be considered as an indicator of the formation of each tissue.

Organ regeneration in vertebrates generally proceeds in a unidirectional manner, from the trunk to the distal part of the organ. Studies on the regeneration polarity of fin rays are limited and it has been suggested that their regeneration is also unidirectional from the proximal to the distal part of the fin [3,25]. One of the studies on the regeneration polarity of fin rays involved the partial excision of fin rays and an observation of the direction of the subsequent regeneration. When a part of the caudal fin ray of the zebrafish is excised, the missing part regenerates from the remaining tissue at the proximal margin of the hole, but not in the opposite direction [3,25]. Although the mechanism of polarized regeneration of the fin ray is not well understood, opposite regeneration of the caudal fin has been reported in long fin (*lof*) mutants of zebrafish [26], and the calcineurin signaling pathway has been reported to be involved in the polarity of caudal fin regeneration [26]. These results suggest that opposite directional regeneration of the zebrafish caudal fin only occurs when there are specific genetic mutations present or under pharmacological conditions. In a classical experiment, fin ray regeneration from the distal edge of the hole was observed using goldfish [27]. Therefore, the fin ray of wild-type zebrafish may also have an ability to regenerate in the opposite direction.

In this study, we re-analyzed the polarity of caudal fin ray regeneration in wild-type zebrafish by examining the regeneration of a hole in the fin. Histological observation revealed that fin ray regeneration progressed unidirectionally from the proximal margin to the distal direction, as previously reported. However, genes induced in the early stage of regeneration either in the wound epidermis or blastema were also expressed in cells in the distal margin, suggesting that an early regenerative response is also induced in the distal margin. To elucidate the cause of unidirectional fin ray regeneration, we analyzed the sheet-like region at the apical half of the regenerated tissue on the proximal margin. The expression of several regeneration-related genes was reduced in the region, which distinguished this region from more inner regions and because no similar sheet-like region was observed at the distal margin of the hole, we hypothesized that this tissue was related to the polarity of the fin regeneration and examined the conditions under which this sheet-like region could form at the distal margin. When the distal margin of the hole was kept out of contact with the proximal margin by manipulation, the sheet-like tissue was autonomously formed in the distal margin, and fin ray regeneration also occurred from the distal margin. These results suggest that the zebrafish fin ray has an ability to regenerate distal structures bidirectionally, but regeneration is usually restricted to the proximal margin associated with the preferential formation of the sheet-like tissue on the proximal margin.

## 2. Materials and Methods

### 2.1. Fish

Zebrafish (AB strain) were purchased from a local supplier and were housed in a recirculating system (14 h day/10 h night cycle; 27 °C). Amputation and regeneration experiments of the caudal fin were performed on 6–12-month-old adult zebrafish. All experimental protocols were evaluated and approved by the Regulation for Animal Research at Tokyo University of Science (approval number: 1880).

### 2.2. Fin Ray Excision

**Figure 1:**
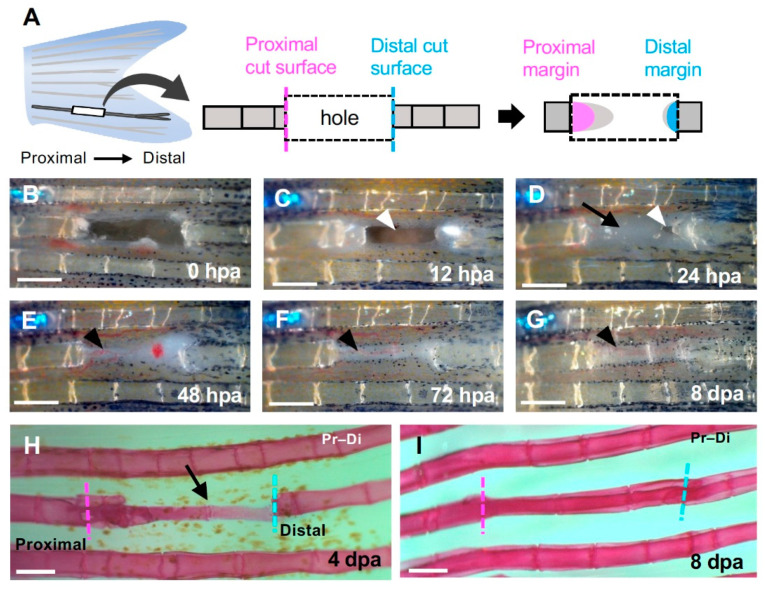
Regeneration of the hole induced by partial excision of the fin ray. (**A**) Schematic diagram of fin ray hollowing. Three to four segments were hollowed out to exclude the branching area (left). The regenerated structures formed from the cells that were distributed on the proximal (magenta) or distal (cyan) surfaces of the hollowed-out hole were investigated (center and right). (**B**–**G**) Time course of regeneration in the hole. (**B**) 0 h post-amputation (hpa); (**C**) 12 hpa; (**D**) 24 hpa; (**E**) 48 hpa; (**F**) 72 hpa and (**G**) 8 days post-amputation (dpa). (**C**) At 12 hpa, the hole remained open (arrowhead). (**D**) At 24 hpa, the hole was almost covered by epithelium (arrow) except for a small portion near the distal surface (arrowhead). (**E**–**G**) At 48 hpa and later, precursor tissue of the fin ray was extended from the proximal side (black arrowheads). (**H**) At 4 dpa, a fin ray primordium was formed from the proximal to distal edge (black arrow). (**I**) At 8 dpa, segments were observed and regeneration was complete. Scale bars = 300 μm.

Zebrafish were anesthetized with tricaine (Tokyo Kasei, T0941). Then, the caudal fin ray and surrounding soft tissue were hollowed out in a rectangular shape using a fine cutter and fine forceps (Figure 1A). In most experiments, 3–4 segments of a single fin ray that did not include the bifurcation were excised. The fin was left to regenerate until the desired time and used for subsequent experiments. In all experiments, the fins were fixed in 4% para-formaldehyde (PFA)/PBS and used for subsequent experiments. Each experiment was independently performed at least three times, and almost the same results were obtained.

To keep the distal edge of the hole separate from the tissues formed in the proximal margin, all epithelium and underlying mesenchyme regenerated from the proximal margin were re-excised at 24 h after the first fin ray excision. This process was repeated two more times so that the distal surface of the hole was kept independent from the proximally derived tissue and allowed to regenerate from distal edge.

### 2.3. Histological Analysis

To analyze the skeletal pattern, the fin ray skeleton was fixed with 4% PFA and dehydrated with methanol. The samples were stained with an alcian blue and alizarin red solution, then cleared with 50% Glycerol-DDW. For the histological sections, the fixed samples were decalcified with EDTA, sectioned with cryostat, and stained with hematoxylin and eosin according to standard procedures.

### 2.4. Whole Mount in Situ Hybridization

All probes used in this study were synthesized using DIG RNA Labeling Mix (Sigma-Aldrich). *msxb* plasmid was provided by Dr Yano (Jikei Medical School, Japan). The *fgf20a*, *fgf24*, *msxd*, and *pea3* were cloned by PCR. Primers used in the study were as follows:

*fgf20a* forward: 5′-ATGGGTGCAGTCGGCGAGCTGGTGG-3′
*fgf20a* reverse: 5′-TCAGCTGTGACCTAGAACATCCTTG-3′
*fgf24* forward: 5′-ATGTCTGTTCTGCCGTCAAGGTTCA-3′
*fgf24* reverse: 5′-TCAGTTTGTATTGGGGTTGGGTT-3′
*msxd* forward: 5′-ATGTCCGCGTCCGCGAGCCTGAAGG-3′
*msxd* reverse: 5′-TCATGCGAGGTGATACATGCTGTA-3′
*pea3* forward: 5′-ATGGATTATAAGATGGATGGATATC-3′
*pea3* reverse: 5′-TTAGTACATGTAGCCTTTGGAGTAGG-3′

Whole-mount in situ hybridizations were performed according to the method described by Sims et al. [28] with minor modifications.

### 2.5. Immunohistochemistry

For the immunocytochemistry, we used the following antibodies. As the primary antibodies: anti-p63 (1:200, #ACR163A, BCM, Funakoshi), anti-BrdU (1:200, MS-1058-P0, Thermo SCIENTIFIC, Waltham, MA, USA), anti-active caspase (1:500, R&D systems, AF835), and anti-tubulin (1:250, Sigma, T6793, St. Louis, MO, USA). As the secondary antibodies, Alexa 488 labeled anti-mouse IgG (1:500, Abcam, #AB150109, Cambridge, UK) and Alexa 488 labeled anti rabbit IgG (1:500, ThermoFisher, #A-11008, Waltham, MA, USA) were used. The detailed process of the immunohistochemistry was performed according to the method described in Ohgo et al. [29].

### 2.6. 3D Reconstruction of the Fin Ray Skeleton

A 3D reconstruction of the regenerated fin ray was performed with the correlative light microscopy and block-face imaging (COMBI) method described in Tajika et al. [30]. Briefly, each alizarin-stained fin ray was embedded in an OCT compound and then sectioned with a cryostat. Each surface of the sectioned plane was photographed, the photo data were stacked, and the image of the fin skeleton was 3D-reconstructed using HOROS (Athens, Greece).

## 3. Results

### 3.1. Morphological and Histological Analysis of Hole Regeneration

First, to show the polarity of the fin regeneration, we hollowed out 3–4 segments of one fin ray to make a hole and analyzed the time course of the hole closure and fin ray regeneration. We compared the morphology of both the proximal and distal margins, and the tissue structures that formed when each margin was repaired (Figure 1A).

Twelve hours after excision, the entire edges of the excised hole were covered by epithelium (Figure 1C). By 24 h after amputation (hpa), a semi-transparent sheet-like tissue expanded from the proximal edge (Figure 1D, arrow), and almost all of the hole except a small portion near the distal edge was filled by the sheet (Figure 1D, arrowhead). Forty-eight hours later, the hole was completely covered with the sheet, and a subcutaneous cell aggregate extended from the proximal side (Figure 1E, arrowhead). After 72 h, a regenerated fin ray primordium was formed from the proximal side (Figure 1F, arrowhead); after eight days, the regenerated fin ray with segments reached the distal end of the excised fin ray (Figure 1G, arrowhead). We also analyzed the shape of the skeleton during regeneration. Four days later, the regenerated fin ray, which was thinner than the original fin ray, was observed from the proximal margin (Figure 1H, arrow); however, no primordium was observed in the distal margin. Eight days later, the fin ray regenerated with several segments, and the proximal and distal fin ray fused (Figure 1I).

**Figure 2:**
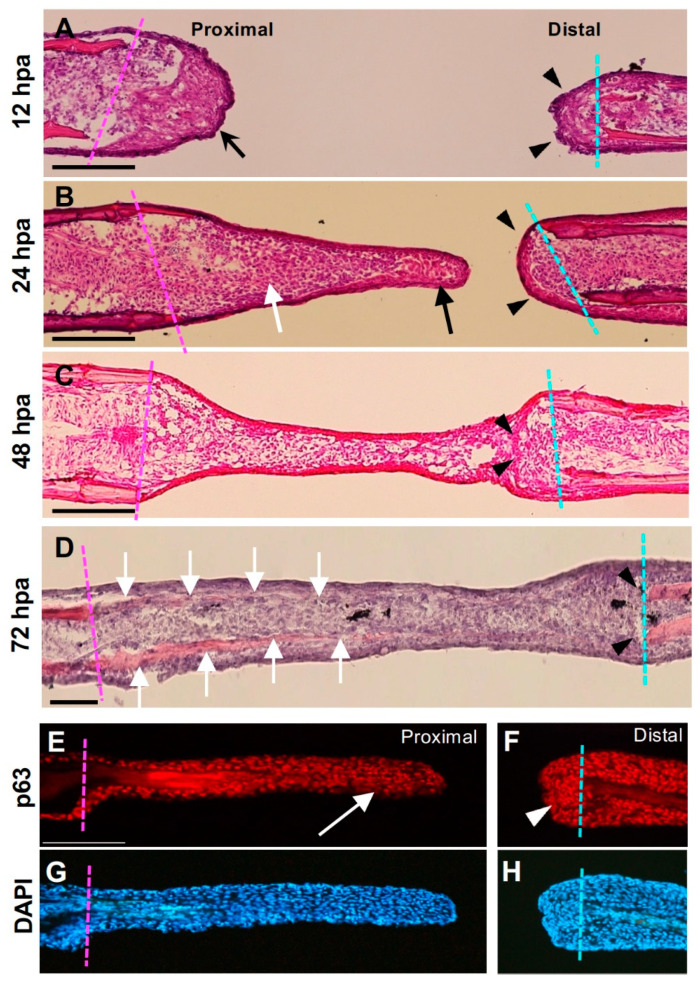
Histological analysis of the hole excision site. (**A**–**D**) Hematoxylin–eosin–stained images after fin ray hollowing. The magenta dotted line indicates the cut surface of the proximal part, and the cyan dotted line indicates the cut surface of the distal part. (**A**) At 12 h post-amputation (hpa), the proximal surface of the hole was covered with thick epithelium (arrow), whereas the distal surface was covered with thin epithelium (arrowhead). (**B**) At 24 hpa, the extended tissue was at the proximal cut surface of the hole. The apical part of the tissue mainly consisted of the epithelial sheet (arrows), and some mesenchymal cells beneath the sheet were also visible. By contrast, the distal cut surface was covered with a thin epithelial sheet and was not elongated (arrowheads). (**C**) The epithelium and mesenchyme extending from the proximal surface of the hole connected with those in the distal surface. At the junction, epithelial-like cellular fragments derived from the distal surface remained (arrowheads). At this stage, bone formation had not occurred. Scale bars = 300 μm. (**D**) At 72 hpa, newly formed bone tissue was observed from the proximal edge toward the tip (arrows), whereas no regenerated bone was observed from the distal cut surface. (**E**) Distribution of the p63 protein in regenerated tissue from the proximal margin. In the apical half of the tissue, there was a layer of p63-positive epithelial cells (arrow). (**F**) Distribution of p63 in the distal margin. Although accumulation of p63-positive cells was visible (arrowhead), they do not extend proximally. (**G**,**H**) are DAPI staining views of (**E**,**F**), respectively.

We then performed a histological analysis. At 12 hpa, both the proximal and distal cut ends were covered by epithelium (Figure 2A). The proximal end was covered with thick epithelium (Figure 2A, arrow), whereas the distal end was covered with thin epithelium (Figure 2A, arrowheads). At 24 hpa, a distally elongated structure was formed on the proximal margin. In the apical part of the structure, the epithelial cell layers formed a somewhat thickened sheet (Figure 2B, arrow), and some mesenchymal cells were also observed beneath the sheet. By contrast, in the distal margin of the hole, the surface was covered with the epithelium, whereas no sheet-like tissue that expanded toward the hole was formed (Figure 2B, arrowheads). At 48 hpa, tissues formed on the proximal and distal margins were connected and no obvious hole-like gap remained (Figure 2C). At the junction of both margins, the remaining epithelial sheet was observed (Figure 2C, arrowheads). At 72 hpa, the proximal and distal edges were fully connected, and regenerated bone tissue was observed from the proximal toward the distal edge (Figure 2D, arrows). In contrast, no bone tissue regeneration toward the proximal edge was observed at the distal edge (Figure 2D, arrowheads).

The distribution of epithelial cells in the apical region of the proximal and distal margins were then examined based on the expression of the p63 protein. At 24 hpa, the apical region of the proximal margin was covered with a p63-positive epithelial cell sheet (Figure 2E, arrow). There were p63-negative mesenchymal cells observed beneath the epithelial sheet near the cut surface, but it was unclear whether mesenchymal cells were distributed in the apical region. In contrast, accumulation of p63-positive epithelial cells was visible in the cut surface of the distal margin, but they did not extend as a sheet-like tissue (Figure 2F).

### 3.2. Expression of Regeneration-Related Genes during Hole Regeneration

**Figure 3:**
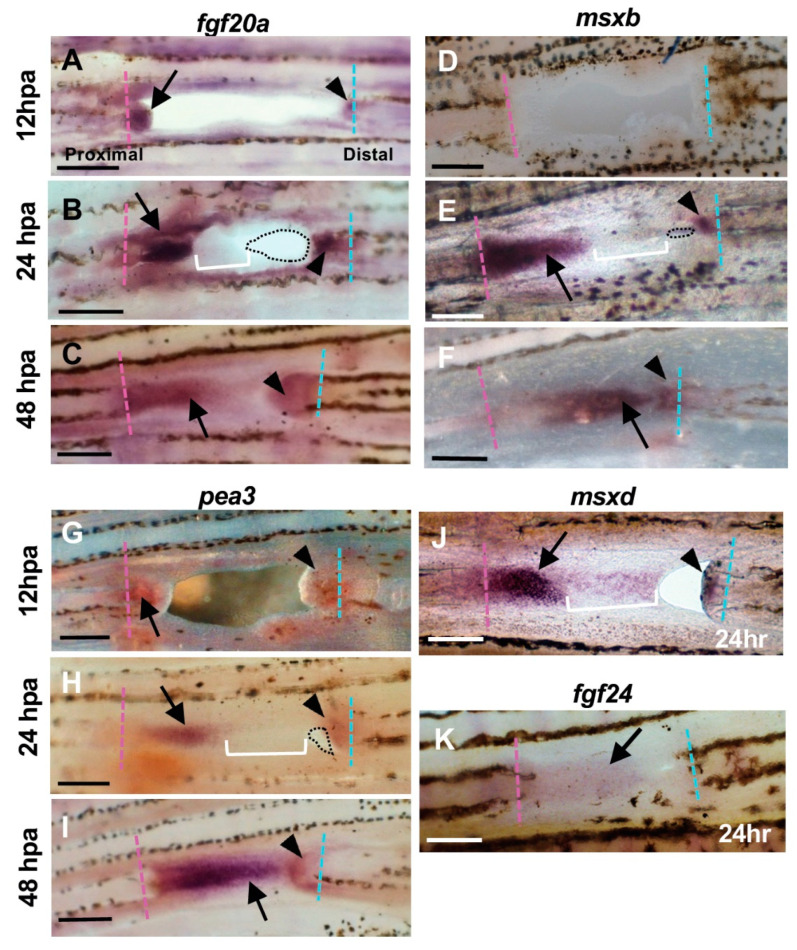
Expression of regeneration-related genes during hole closure. (**A**–**C**) *fgf20a* expression. (**A**) At 12 h post-amputation (hpa). The *fgf20a* expression was observed in the cells of the proximal margin (arrow), and distal margin (arrowhead) of the hole. (**B**) At 24 hpa. In the proximal margin, *fgf20a* was strongly expressed in the cells of the inner half of the regenerated tissue (arrow), whereas the expression was faint in the apical half of the tissue (bracket). In the distal margin, *fgf20a* expression was also observed, but the area of expression was small (arrowhead). Dotted lines show the remaining hole. (**C**) At 48 hpa. The *fgf20a* expression was maintained in both proximal- and distal-derived tissues (arrow and arrowhead, respectively), and both domains were close together. (**D**–**F**) *msxb* expression. (**D**) At 12 hpa, *msxb* was not expressed. (**E**) At 24 hpa, in the proximal margin, *msxb* was strongly expressed in the cells near the cut surface of the regenerated tissue (arrow). The expression was faint in the distal half of the tissue that filled the hole (bracket). In the distal margin, *msxb* expression was restricted to a small area (arrowheads). Dotted lines show the remaining hole. (**F**) At 48 hpa, *msxb* was maintained in both proximal- and distal-derived tissues (arrow and arrowhead, respectively). (**G**–**I**) *pea3* expression. (**G**) At 12 hpa, *pea3* expression was observed in the cells both in the proximal and distal margins of the hole (arrow and arrowhead, respectively). (**H**) At 24 hpa, *pea3* was expressed in the cells of the inner half of the regenerated tissue in the proximal margin (arrow), whereas the expression was weakened in the apical half of the tissue (brackets). In the distal margin, *pea3* expression was also observed, although the expression domain was small (arrowhead). Dotted lines show the remaining hole. (**I**) At 48 hpa, *pea3* expression was maintained in both proximal- and distal-derived tissues (arrow and arrowhead, respectively). (**J**) *msxd* expression at 24 hpa. In the proximal margin, *msxd* was strongly expressed in the cells of the inner half of the regenerated tissue (arrow), whereas the expression was weakened in the apical half of the tissue (bracket). In the distal margin, *msxd* expression was also observed, but the area of expression was restricted (arrowhead). (**K**) *fgf24* expression at 24 hpa. The *fgf24* was weakly expressed in the epithelium of the regenerated tissue from the proximal margin (arrow). Expression in the distal margin was unclear. Scale bars = 300 μm.

Next, we examined the gene expression during the hole regeneration. First, we examined the *fgf20a*, which was expressed in the blastema cells during fin regeneration [23,24]. At 12 hpa, *fgf20a* was observed in both the proximal and distal margins of the hole (Figure 3A). At 24 hpa, the *fgf20a* expression was maintained in the cells of the inner half of the proximal-derived tissue (Figure 3B, arrow), but it was faint in the apical half of the tissue (Figure 3B, bracket). Interestingly, *fgf20a* was also expressed in cells of the distal margin (Figure 3B, arrowhead). At 48 hpa, *fgf20a* was expressed in the tissues elongated from the proximal cut surface (Figure 3C, arrow), and the expression at the distal margin was also maintained (Figure 3C, arrowhead).

We also investigated the expression of *msxb,* which is another blastema marker [21,22]. Expression of *msxb* was detected at 24hpa; strong expression of *msxb* was observed in the inner half of the proximal-derived tissue (Figure 3E, arrow), but faint expression was detected in the apical half of the tissue (Figure 3E, bracket). At the distal margin, *msxb* was also expressed in mesenchymal cells (Figure 3E, arrowhead). The expression was spatially restricted but obvious, which indicated the existence of blastema-like cells in the distal margin. At 48 hpa, *msxb* was maintained in the tissues of both the proximal and distal margins (Figure 3F, arrow and arrowhead).

We then examined the *pea3* expression that is mainly expressed in the wound epidermis and its basal layer [20,23]. At 24 hpa, *pea3* expression was observed in the inner half of the proximal-derived tissue, which is where *fgf20a* and *msxb* were also expressed (Figure 3H, arrow and arrowheads), and a reduced expression in the apical half of the proximal-derived sheet-like tissue was also observed (Figure 3H, bracket). The *pea3* expression sites were similar to those of *msxb* and *fgf20a*, but there was a marked decrease in expression in the apical half of the proximal margin at 24 hpa.

We also analyzed the expression of *msxd* and *fgf24* in the wound epidermis [18,19,20,21,22]. The *msxd* was strongly expressed in the inner half of the regenerating tissue on the proximal margin (Figure 3J, arrow), but it was weakly expressed in the apical half (Figure 3J, bracket). In the distal margin, an apparent signal of *msxd* expression was observed (Figure 3J, arrowhead). The *fgf24* expression was weakly observed throughout the regenerated tissue (Figure 3K, arrow).

Overall, the features of regeneration-related gene expression are summarized as follows. In the tissue regenerated from the proximal margin, the expression of regeneration-related genes was apparent in its inner half, whereas the expression in its apical half tended to be faint and this indicates regional differences of the tissue. By contrast, in the tissue of the distal margin, the expression of these genes was also observed despite the failure of subsequent fin ray regeneration.

### 3.3. Cellular Responses during Hole Regeneration

**Figure 4:**
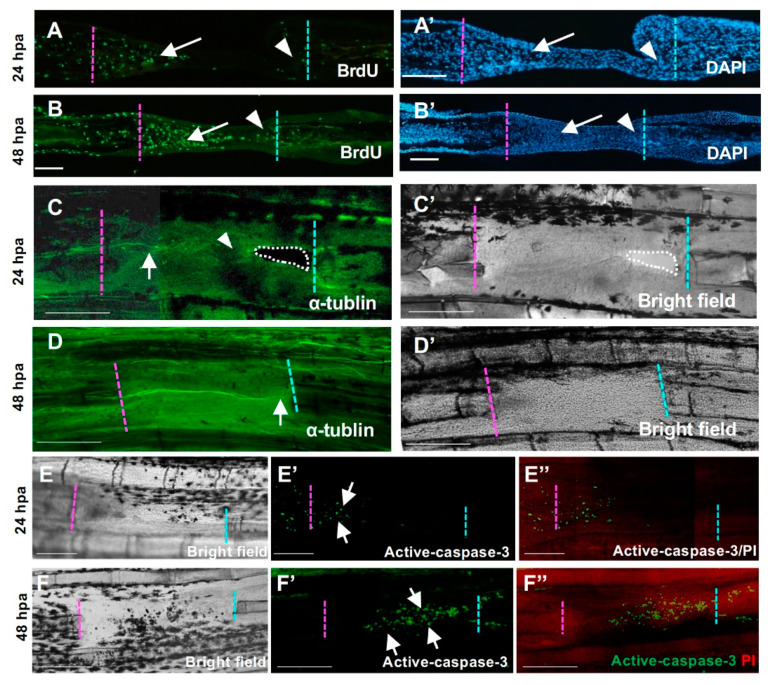
Cellular responses during hole regeneration. (**A**,**A’**,**B**,**B’**) BrdU-positive cells during hole regeneration. (**A**) At 24 h post-amputation (hpa), high proliferation of mesenchymal cells was observed in the proximal-derived tissue (arrow), but few cells were observed in the distal side (arrowhead). (**B**) At 48 hpa, high proliferation of mesenchymal cells was maintained, and proliferation of epithelial cells was also detected. In the distal tissue, some cells were also BrdU-positive (arrowhead). (**A’**) and (**B’**) are DAPI staining of (**A**,**B**), respectively. Scale bars = 100 μm. (**C**,**C’**,**D**,**D’**) Nerve distribution during hole regeneration. (**C**,**C’**) 24 hpa. (**C′**) is the bright field view of (**C**). The nerve fiber extended from the proximal end to the regenerative tissue (arrow). By contrast, few nerve fibers were detected in the apical half of the epithelial sheet and the distal cut end (arrowhead). (**D**,**D’**) 48 hpa. (**D’**) is the bright field view of (**D**). Several nerve fibers extended from the proximal cut end to the distal cut end (arrow). Scale bars = 300 μm. (**E**,**E’**,**F**,**F’**) Apoptosis during hole regeneration. (**E**,**F**) Bright field. (**E’**,**F’**) Active caspase-3. (**E”**,**F”**) Merged images of active caspase-3 and propidium iodide (PI) staining. (**E**,**E’**,**E”**) At 24 hpa, apoptosis was only observed in the epithelium covering the blastema formed on the surface of the proximal side (arrow, *n* = 3). (**F**,**F’**,**F”**) At 48 hpa, apoptotic cells were observed in the tissue derived from the proximal surface (arrow, *n* = 3). Resection edges (dotted lines). Scale bars = 100 μm (**A**,**B**), 300 μm (**C**,**D**).

We next investigated the cell proliferation around the hole by detecting cells incorporating BrdU. At 24 hpa, cell proliferation increased in the proximal margin (Figure 4A, arrow), but was low in the distal margin (Figure 4A, arrowhead). This tendency was also maintained at 48 h. At 48 hpa, proliferation was also high in the proximal side (Figure 4B, arrow), but low in the distal side (Figure 4B, arrowhead), which showed that cell proliferation was preferentially enhanced in the proximal margin.

We further investigated the distribution of nerve fibers at the cut surface because nerve fibers have been suggested to have a role in pectoral fin regeneration [23]. At 24 hpa, nerve fibers were distributed to the proximal half of the regenerated tissue, which coincided with the area where expression of regeneration-related genes was apparent (Figure 4C, arrow). By contrast, few nerve fibers were observed in the apical half of the tissue (Figure 4C, arrowhead). At 48 hpa, nerve fibers extended distally and reached the distal cut surface (Figure 4D, arrow). Therefore, it seems that the promoted cell proliferation and nerve fiber distribution are related to the proximal-biased fin ray regeneration.

We also investigated apoptosis in the regenerating surface of the hole (Figure 4E,F). At 24 hpa, apoptotic cells were observed in the tissue near the cut surface of the proximal margin (Figure 4E,E’,E”, arrows). By contrast, the apoptotic cells were not obvious in the distal margin (Figure 4E,E’,E”). After 48 h, apoptotic cells were observed near the center of the closed hole (Figure 4F,F’,F”, arrows), which coincides with the area occupied by the proximal margin-derived cells. Thus, apoptosis may not be a cause for the failure of regeneration from the distal margin.

These results show that gene expression can be induced in the distal margin and in the proximal margins following fin ray excision, whereas cell proliferation and fin ray regeneration do not progress in the distal margin.

### 3.4. Fin Ray Regeneration from the Distal Margin Can Be Induced by Keeping the Margin Independent from the Proximal Margin

**Figure 5:**
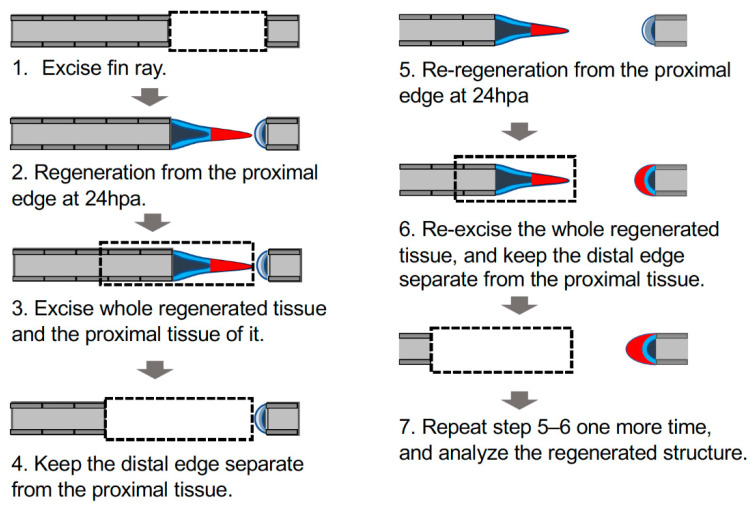
Schema of manipulation to investigate autonomous sheet-like tissue formation in the distal margin. Schema of the experimental procedure; the detailed procedure is described in the Materials and Methods section. Dashed rectangles indicate excision sites of the fin ray; gray, fin ray; light blue, epithelium of the cut surface; dark blue, mesenchyme of the cut surface; red, the sheet-like tissue formed on the proximal margin (triangle) or an equivalent structure on the distal margin (crescent shape).

The results have so far revealed that the cells in the distal margin of the hole can respond to fin ray excision by retaining the ability to regenerate, although the regeneration from this margin did not occur. We considered that one reason for the lack of regeneration in the distal margin may be a failure to form the sheet-like region in the distal ends. We hypothesized that the formation of the sheet-like tissue at the distal edge might take a longer time than at the proximal edge. To test this, we performed a manual manipulation in which the distal and proximal margins were kept separate so that they did not make contact. We excised the sheet-like region formed on the proximal margin three times. The distal end was left in place during this process. After this, regeneration was promoted from the distal and proximal margins, and gene expression at the regeneration site and the morphology of the subsequently formed fin rays were analyzed (Figure 5).

**Figure 6:**
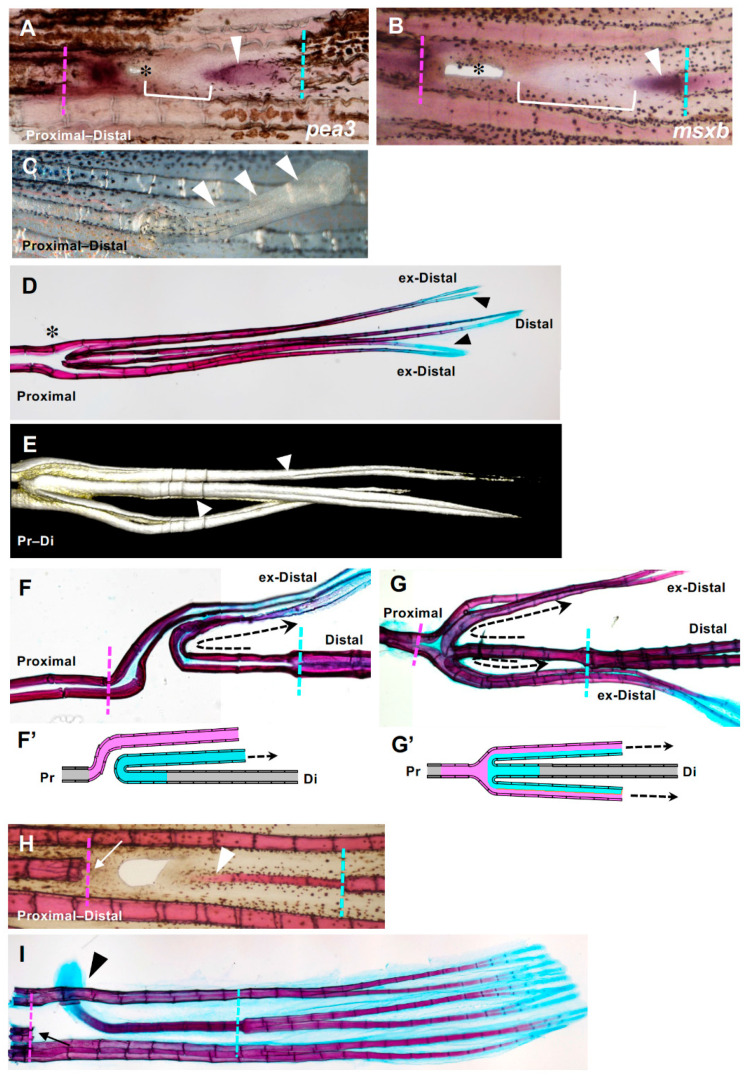
Fin ray regeneration of fin ray from the distal margin of the hole. (**A**,**B**) Strong expression of both *msxb* and *pea3* was observed at both the proximal and distal sides. Arrowheads indicate gene expression that was extended from the distal side to the proximal side. In addition, a distal margin-derived sheet-like tissue was observed (yellow brackets). (**C**) Seven days after excision, a rod-like lateral protrusion was formed from the position of the hole closure (arrowheads). (**D**) An example of the skeletal pattern that was fully regenerated from a hole-excised caudal fin. The original and regenerated fin rays are shown. Regenerated fin rays laterally protruded from the position of the hole with extra branching (asterisk). The distal ends of the regenerated fin rays (indicated as ex-distal) reached the same length as the original fin ray and were stained with alcian blue (arrowheads). (**E**) A 3D reconstruction of the regenerated extra fin ray shown in (**D**) using the COMBI method. Bifurcation of the regenerated fin ray was observed (arrows). (**F**,**G**) Examples of the skeletal pattern at the branching position of the hole-excised caudal fin. The direction of the regenerated fin ray extending from the distal margin is indicated with dotted arrows. (**F’**) and (**G’**) are schema that represent the fin ray patterns shown in (**F**,**G**), respectively. In the schema, fin rays regenerated from the distal margin are indicated in blue, and those regenerated from the proximal margin are indicated in pink. In (**F**) the fin ray that regenerated from the distal margin protruded to either side of the fin at the position where it collided with the fin ray that regenerated from the proximal margin, and then bent distally (*n* = 3/9). In (**G**) a hemi-ray of the distal-derived fin ray and that of the proximal-derived fin ray merged to form one fin ray and protruded to both sides of the fin (*n* = 6/9). (**H**,**I**) A fin ray regenerated from a distal margin after prolonged and repeated excision of tissue at the proximal margin of the hole. (**H**) After one week, the regenerated fin ray was formed in the same plane of the original fin rays and as a continuous structure of the proximal edge of the original fin ray (arrowhead, *n* = 3). (I After 2 weeks of regeneration, the distal end of the regenerated fin ray protruded laterally (arrowhead, *n* = 3). The distal end was stained with alcian blue (arrowhead), as was the original fin ray. During the regeneration process from the distal cut edge, regeneration from the proximal margin did not occur because of repeated excision (arrows).

By this repeated excision, the distal margin remained independent and unaffected by the proximal end for 96 h. At 24 h after the final manipulation, *msxb* and *pea3* were expressed at both the proximal and distal ends (Figure 6A,B); in particular, their expression at the distal end was enhanced and extended (Figure 6A,B, arrowheads), compared with the expression observed in the simple hole regeneration. In addition, a new sheet-like region in which there was faint expression of *msxb* and *pea3* was also observed (Figure 6A,B, brackets). The hole was located proximal to the tissue (Figure 6A,B, asterisks), suggesting the sheet-like region was formed from the distal edge and expanded proximally to fill the hole.

We then followed the subsequent regeneration of the fin rays. In the first week, a rod-like fin ray was observed protruding from the corresponding position of the hole (Figure 6C, arrowheads). The protruding rod-like fin rays were elongated distally until they reached the same length as the distal end of the original fin rays in the following two weeks (Figure 6D, arrowheads).

Next, we analyzed the skeletal pattern by comparing the regenerated and original fin ray. In the position where a lateral protrusion of fin ray was observed, extra branching of fin rays was observed (Figure 6D, asterisk). As mentioned later, the branching pattern was categorized into two groups. In the distal end of each regenerated fin ray, alcian blue-positive tissues were observed (Figure 6D, arrowheads), as in the distal end of the original fin rays. By 3D reconstruction of the fin ray with the COMBI method [30], we confirmed that distal-derived fin rays also bifurcated and then bent distally, which was similar to the normal fin rays (Figure 6E, arrowheads).

We then investigated the connection between the regenerated fin rays and the original fin rays (Figure 6F,F’,G,G’). We categorized the regenerated fin rays into two groups. In the first group, the distal-derived regenerated fin ray first extended proximally but then protruded laterally where the distal-derived fin ray met the proximal-derived fin ray and turned back to extend distally to the caudal fin (Figure 6F,F’, arrows). The elongation was often parallel to that of the proximal-derived fin rays. In the second group, each hemi-ray of distal edge-derived fin rays bent toward opposite sides of the fin, and each merged with the hemi-rays of the fin ray from the proximal side; they then formed a single fin ray structure (Figure 6G,G’, arrows). In this experiment, the second group was dominantly formed (*n* = 3/9 for the first group, *n* = 6/9 for the second group).

These results indicate that fin ray regeneration can also occur from the distal margin, and the “distal end” of the regenerated fin ray from the distal margin of the hole has the same structure as the distal end of the original fin ray; however, it is possible that the proximal tissue still affected the distal tissue and induced regeneration if they made contact after we had excised the tissue three times. Therefore, to eliminate this possibility, we attempted to make the distal margin independent for a longer period by repeated excision of the sheet-like tissue of the proximal margin for another two weeks. Because of the repeated excision, regeneration from the proximal margin did not occur during the experiment (Figure 6I,H, arrows). After one week, a thin but apparent fin ray from the distal margin was formed (Figure 6H, arrowhead, *n* = 3). At this stage, the regenerated fin ray was in the same plane as the original fin rays and there was continuous regeneration of the distal edge of the original fin ray (Figure 6H, arrowhead). After two weeks of regeneration, the distal end of the regenerated fin rays elongated more and protruded laterally (Figure 6I, arrowhead, *n* = 3). The “distal end” of the regenerated fin ray from the distal margin of the hole was stained with alcian blue like the distal end of the original fin ray (Figure 6I, arrowhead). These results show that regeneration of the fin rays from the distal margin of the hole occurs independently of regeneration from the proximal margin, and they also indicate that cells at the distal edge of the hole could regenerate the fin rays but are restricted to the more distal structures.

Taken together, these results indicate that the fin ray has an ability to regenerate bidirectionally, but regeneration is likely to be limited to the proximal margin under normal conditions, because of the preferential formation of a sheet-like tissue on the proximal margin.

## 4. Discussion

In this study, we investigated the polarity of caudal fin regeneration in zebrafish by focusing on the hole closure following fin ray regeneration. Our finding are as follows. First, even though fin regeneration proceeds in a unidirectional manner from the proximal margin of the hole in normal regeneration, expression of regeneration-related genes was also induced in the distal margin of the hole. Second, a sheet-like tissue was formed in the proximal margin of the hole, but not in the distal margin. Finally, if the distal margin was kept separate from the proximal margin, the sheet-like tissue was also formed in the distal margin and ectopic regeneration of the fin ray occurred. From these results, we concluded that cells in the distal margin also have an ability to regenerate fin ray autonomously, the same as cells in the proximal margin.

### 4.1. Fin Regeneration Proceeds Unidirectionally from the Proximal Side, but the Regenerative Response also Occurs in the Distal Side

Our results indicate that, although fin ray regeneration proceeds in one direction from the proximal margin of the hole to the distal direction, the initial response to regeneration occurs in both the proximal and distal margins. Histological analysis showed that repair of the hole and fin ray regeneration proceeded in a unidirectional manner from the proximal to distal margin, as in previous reports (Figure 1); however, the regeneration marker genes that we investigated were expressed in both the proximal and distal margins (Figure 3). The expressions in the distal margin were restricted to a smaller area than at the proximal margin (Figure 3); thus, the response in the distal margin may be limited compared with the normal response at the proximal margin. In a fracture model of fin rays, bone fracture repair was initiated in both the proximal and distal sides [31], indicating that local tissue or organ repair proceeded from both directions. Cellular and tissue behaviors during the hole regeneration observed in our study may be reflected in these responses.

In contrast to our results, a recent report by Cao et al. [26] on fin hole regeneration did not mention the expression of regeneration-related genes at the distal margin of the hole. This is possibly due to the differences in experimental conditions; in their report, two fin ray rows were excised, but we excised one row. In addition, the length of the hole prepared in our study was longer than that in their experiment [26]. Because the area of gene expression in the distal margin was restricted to a small area in our experiment, as mentioned above, the signals of gene expression may be unclear in other experimental conditions.

Even though a regenerative response was also occurring at the distal margin in terms of the expression of several regeneration-related genes, the fin ray did not regenerate at the distal margin during hole regeneration in the absence of experimental manipulation (Figure 1, Figure 2 and Figure 3). This result indicates that the cells at the distal margin of the hole maintain their regenerative ability, but under normal regenerative conditions, it is not sufficient to progress the regeneration. During fin regeneration, the proliferation of cells is induced both in the epithelial cells and blastemal cells [12,13,19,32]. In our experiment, less proliferation of cells was induced in the distal margin in contrast to the proximal margin where increasing proliferation was induced (Figure 4A,B). This indicates that that gene expression is not always linked to the induction of cell proliferation and the following regeneration, and again suggests that this sheet-like tissue is necessary to for regeneration to proceed (see below).

### 4.2. Role of the Sheet-like Tissue in Fin Ray Regeneration and Polarity

Our results in the present study indicate that the formation of sheet-like tissue is important for fin regeneration in hollowed-out holes and is strongly related to regeneration polarity. Previous studies of hole regeneration in the fin mainly described the morphology of regenerating fin ray [3,25], but tissue-level analysis around the hole was not performed. Cao et al. [26] investigated gene expression during the regeneration of excised fin rays but did not mention the expression of regeneration-related genes in the distal edge in wild-type fish [26]. In addition, there has been no mention of the sheet-like region formed on the cut surface; however, Murciano et al. [33] studied inter-ray wound healing and subsequent regeneration, and reported the formation of a membranous, epithelial ‘meniscus-like edge’ where no gene expression was observed [33]. In our study, we focused on the sheet-like tissue formed at the proximal margin, and this may correspond to the membranous structure described in Murciano et al. [33]. Our results and those of Murciano et al. [33] indicate that this sheet-like tissue becomes apparent during the fin regeneration of a confined area along the proximodistal axis.

The formation of this tissue may be associated with wound repair in confined areas; however, as we have shown, the formation of the sheet-like tissue on the distal side of the hole seems to require time; therefore, the sheet-like tissue is not simply formed for wound repair. Rather, our results suggest that its formation is closely related to the regeneration and the polarity of the fin ray. In the usual fin regeneration experiment, in which the distal end of the fin is cut off, a structure that spreads in the regeneration direction, similar to the sheet-like tissue observed in our experiment, is not observed. Instead, the cut surface is covered with a slightly thickened epithelial sheet, the wound epithelium [3,5,7,8]. Its importance in fin regeneration has been previously mentioned [3,5,7,8]. During normal regeneration, it is quite possible that functionally homologous cells or tissues, although not in the form of expanded sheets, arise in the wound epithelium, and that this promotes subsequent regeneration.

### 4.3. Polarity of Fin Ray Regeneration

We found that cells at the distal margin of the hole could regenerate fin rays when the distal margin was kept independent from the proximal margin (Figure 6). In this case, a sheet-like region was also formed at the distal end, although it took longer (Figure 6). Thus, in wild-type zebrafish, the distal margin was also capable of autonomously regenerating fin rays under specific conditions. This is reasonable because regeneration-related genes were expressed at the distal margin (Figure 3); however, in previous reports [3,25,26], and even in our results (Figure 1), spontaneous regeneration of the hole did not result in regeneration of the fin rays from the distal margin. Taken together, these results suggest that regeneration from the distal margin may be suppressed under spontaneous regeneration. One possible explanation is that cells of the distal margin do not regenerate if the proximal and distal tissues are in a continuous plane or regeneration field. In this case, there may be an inhibitory effect on regeneration from the tissue at the proximal margin toward the distal margin, but the specific mechanism is not known. If such a system exists, it may be useful to prevent excessive regeneration of unnecessary structures from the distal margin during spontaneous regeneration.

In a classic study, regeneration from the distal margin was reported in a fin ray excision experiment of the goldfish tail fin by Nabrit [27]. In the report, four fin rays and six segments in the caudal fin were excised, and the fin rays regenerated from both the distal and proximal margins of the hole. The fin rays that regenerated from the distal margin protruded laterally from the fin to form ectopically elongated fin rays, which corresponded to our observation. Based on our results, Nabrit’s [27] results can be interpreted as follows: it required time for the holes in the caudal fin to be closed, resulting in the induction of regeneration from the distal end. Cao et al. [26], however, reported that large holes in the zebrafish tail fin did not induce regeneration from the distal margin. Therefore, there may be some species-specific differences in hole regeneration between zebrafish and goldfish.

### 4.4. Factors That Influence Regeneration Polarity

Our results show that the unidirectional regeneration of fins reflects the polarity of the sheet-like tissue formation, although the mechanism of polarized formation is unclear. The regeneration of zebrafish pectoral fins involves the distribution of nerve fibers [23]. Hole regeneration in mouse auricles has also been noted to have regeneration polarity along the proximal–distal axis that was suggested to be related to nerve distribution [34,35,36]. Although the present results do not confirm neural dependency in the early stages of the sheet-like tissue formation, further investigation of the relationship with neural distribution is needed.

A relationship between the unidirectional hole regeneration and calcineurin activity has been suggested [26]; in that report, inhibition of calcineurin activity could induce the regeneration of fin rays from the distal margin in wild-type zebrafish. Although the relationship between calcineurin activity and sheet-like tissue formation is unclear, it is possible that keeping the distal margin independent reduces the calcineurin activity and leads to sheet-like tissue formation.

### 4.5. Conclusions

In this study, we showed that regeneration of the zebrafish caudal fin ray proceeds in a unidirectional manner from the proximal margin, and that the sheet-like tissue, which is formed on the proximal margin, must be understood as an initial stage of ray regeneration potential determining the direction of the regeneration. Cells in the distal margin have an ability to regenerate fin rays by forming a sheet-like tissue, but this ability seems to be suppressed during spontaneous regeneration. The formation of this sheet-like tissue is usually unidirectional from the proximal margin and takes time to form at the distal margin. Therefore, studying the formation of this tissue and its function may provide a new perspective on why regeneration proceeds in one direction.

In wound healing and organ regeneration research, understanding tissue polarity is important for promoting stable healing and regeneration. Further studies of fin repair and subsequent regeneration polarity are expected to provide necessary information for elucidating regeneration polarity in other organs.

## Data Availability

Not applicable.
